# Squamous-cell carcinoma arising in a pilonidal sinus: case report and review of the literature

**DOI:** 10.1186/1471-2318-11-S1-A28

**Published:** 2011-08-24

**Authors:** Antonio Martino, Ciro De Martino, Anna Pisapia, Gautam Maharajan, Marco Evangelista

**Affiliations:** 1 Casa di Cura “A. Grimaldi” di San Giorgio a Cremano (NA). Dipartimento di Chirurgia, Italy; 2 Università degli Studi di Napoli “Federico II”. U.O.C. di Chirurgia Generale, Italy

## Background

Squamous-cell carcinoma arising in a pilonidal sinus is a rare complication of a common disease. More than 60 cases are reported in literature. In all patients, squamous carcinoma arises in a long-term pilonidal disease. The gold-standard for treatment is radical excision of the neoplasm, with tumour-free margins. Some Authors consider the effectiveness of postoperative chemo- or radiotherapy, to reduce local recurrence.

## Methods

Authors report the case of a 65-years old male, with a 15-year history of recurrent pilonidal disease. He was admitted to the General Surgery Unit because of the development of a bleeding ulcerated lesion in the pilonidal area. The patient underwent an incisional biopsy (showing squamous-cell carcinoma), preoperative staging with total body CT-scan, and finally radical surgery. The large wound healed by secondary intention. Definitive histology confirmed diagnosis and revealed tumour-free margins. After being discharged, the patient was followed up on as an outpatient.

## Results

Hospital stay took 10 days, due to the complexity of the first medications. A posterior rectocele was observed, but no functional diseases were identified. The complete formation of the scar took three months. After six months, no complication or signs of recurrence were observed.

## Conclusions

Squamous-cell carcinoma arising in a pilonidal sinus is a rare occurrence that develops only in long-standing pilonidal diseases. The tumour grows very slowly, but has a high local invasivity. Inguinal lymphadenopaties at the time of diagnosis is related to a poor prognosis. Local recurrence is usual and early. Enlargement of excision in case of local recurrence, and abdomino-perineal resection if sphincters are involved, may improve survival rates. There are not enough trials to assess the usefulness of preoperative or postoperative chemo- and radio- therapy.
